# In Vitro Magnetic Techniques for Investigating Cancer Progression

**DOI:** 10.3390/cancers13174440

**Published:** 2021-09-03

**Authors:** Sarah Libring, Ángel Enríquez, Hyowon Lee, Luis Solorio

**Affiliations:** 1 Weldon School of Biomedical Engineering, Purdue University, West Lafayette, IN 47907, USA; slibring@purdue.edu (S.L.); aenrique@purdue.edu (Á.E.); 2 Birck Nanotechnology Center, Purdue University, West Lafayette, IN 47907, USA; 3 Center for Implantable Devices, Purdue University, West Lafayette, IN 47907, USA; 4 Purdue Center for Cancer Research, Purdue University, West Lafayette, IN 47907, USA

**Keywords:** magnetism, cancer, tumor, in vitro, metastatic cascade, review

## Abstract

**Simple Summary:**

This review focuses on the advantages achieved by incorporating magnetic forces into culture platforms used to study cancer progression in the laboratory. Due to the complex interactions that occur between cancer cells and their environment throughout primary tumor growth and metastatic spread, benchtop techniques are essential for decoupling these factors at several stages of disease progression where traditional animal models would fail. Breakthroughs in our understanding of cancer biology and mechanics through these benchtop techniques can ultimately lead to better-designed precision medicine platforms and clinical therapeutics for patients.

**Abstract:**

Worldwide, there are currently around 18.1 million new cancer cases and 9.6 million cancer deaths yearly. Although cancer diagnosis and treatment has improved greatly in the past several decades, a complete understanding of the complex interactions between cancer cells and the tumor microenvironment during primary tumor growth and metastatic expansion is still lacking. Several aspects of the metastatic cascade require in vitro investigation. This is because in vitro work allows for a reduced number of variables and an ability to gather real-time data of cell responses to precise stimuli, decoupling the complex environment surrounding in vivo experimentation. Breakthroughs in our understanding of cancer biology and mechanics through in vitro assays can lead to better-designed ex vivo precision medicine platforms and clinical therapeutics. Multiple techniques have been developed to imitate cancer cells in their primary or metastatic environments, such as spheroids in suspension, microfluidic systems, 3D bioprinting, and hydrogel embedding. Recently, magnetic-based in vitro platforms have been developed to improve the reproducibility of the cell geometries created, precisely move magnetized cell aggregates or fabricated scaffolding, and incorporate static or dynamic loading into the cell or its culture environment. Here, we will review the latest magnetic techniques utilized in these in vitro environments to improve our understanding of cancer cell interactions throughout the various stages of the metastatic cascade.

## 1. Introduction

In 2021, there will be almost 1.9 million newly diagnosed cancer cases and over 600,000 cancer deaths in the United States [1]. Worldwide, there are currently around 18.1 million new cases and 9.6 million cancer deaths yearly [2]. It is clear that advances in the diagnosis and treatment of cancer remain a high priority in biological, healthcare, and engineering research disciplines. Several advances for both cancer treatment and basic research are achieved through the incorporation of magnetic technologies. For example, the untethered transmission of force attainable through magnetic force allows for remote access, facilitates targeted delivery and precise movement in vivo and in vitro, and enables the easy sorting of specific cell types. In this review, we will first give an overview of primary tumor growth and metastatic progression (Section 2) and the principles of magnetic transduction (Section 3). We will then briefly discuss recent advances using magnetic techniques for in vivo and ex vivo patient care (Section 4) before focusing on in vitro magnetic platforms as an invaluable supplement to in vivo animal models in improving our understanding of the complex interactions that occur in cancer biology (Section 5 and Section 6).

## 2. Primary Tumor Growth and Metastatic Progression

In order for a solid carcinoma to grow and eventually metastasize, cells of a particular tissue must first acquire features that enable aberrant survival and rapid division and then must acquire additional hallmark features that enable local movement and systemic spread throughout the body. The growth of the primary tumor and the metastatic cascade can be broken into four main categories: primary tumor growth, invasion, survival in circulation, and overt metastasis [3]. In the first step, cancer cells evade antiproliferative and apoptotic signals typical of a tissue in homeostasis [4]. A permissive tumor microenvironment is orchestrated by the recruitment and reprogramming of cancer-associated fibroblasts and other stromal cells which foster angiogenesis and alter the extracellular matrix (ECM) content and architecture [5,6]. As the primary tumor develops, premetastatic niches also develop throughout the body, fueled by extracellular vesicle communication from the cancer cells. Changes to the ECM, suppression of the immune system, and an increase in vascularization to increase nutrient transport prime distant areas of the body to be amenable to the cancer cells upon arrival [7,8,9,10,11,12].

As epithelial cancer cells continue to lose their apical-basal polarity, weaken cell-cell junctions, and rearrange their cytoskeleton, invasive capacities are acquired in a subset of the population. This is referred to as the epithelial to mesenchymal transition (EMT). EMT is a common process utilized in embryonic development and tissue regeneration. This developmental pathway is reactivated, although typically incompletely, in many cancer types [13,14]. EMT is initialized by both internal transcription factors (e.g., the Snail, Twist, and Zeb families) and external microenvironmental cues, such as increased fibrosis and inflammation. EMT in cancer is characterized by the induction of these EMT-transcription factors, the loss of epithelial gene products and the gain of mesenchymal gene products (e.g., loss of E-cadherin and gain of vimentin), and the acquisition of a mesenchymal cell phenotype (i.e., spindle-shaped, migratory, loss of cell–cell cluster packing) [13]. Tumor cells with this mesenchymal phenotype migrate through the basement membrane and invade the stroma toward blood and lymphatic vessels by utilizing rearranged bundles of collagen and fibronectin that lead radially outward from the tumor edge [15].

After intravasation, cancer cells must withstand the shear forces of the vasculature. It is estimated that <0.02% of circulating tumor cells (CTCs) complete metastasis [16,17]. Tumor cells may circulate individually or they may form circulating emboli when small clusters of cells maintain intracellular junctions and intravasate together [15,18]. Clusters can provide a survival advantage by shielding internal CTCs from fluid shear stress and immune assault [19]. Additionally, heterotypic clusters, which include CTCs and additional cell types, such as neutrophils and/or cancer-associated fibroblasts, appear to be rare in peripheral circulation but seem to possess a significant metastatic advantage over other common CTC arrangements [19,20].

CTCs must eventually extravasate into new surrounding tissue. To do so, CTCs arrested in small capillaries adhere to the endothelium, transmigrate, and invade the stromal matrix [21]. Disseminated cells from various cancers preferentially reside in different organs, a feature known as organotropism. This non-random distribution is not accounted for by simple circulation patterns, and instead seems dictated by numerous additional factors including tumor-intrinsic factors and organ-specific niches [22,23,24]. Once at secondary sites, disseminated cancer cells may lay dormant until external stimuli are presented. Some stimuli found in recent research include further adjustments to the niche matrix, neutrophil extracellular traps induced by inflammation, and manipulation of tumor cell metabolic pathways [7,22,23,25] These mechanical and biochemical signals allow the cancer cells to undergo mesenchymal to epithelial transition, at which point a secondary tumor, fueled by rapidly proliferating epithelial cancer cells, develops in a similar manner to the primary tumor [25]. Although originally believed to be a linear process, wherein metastasis was the final product of a primary tumor outgrowing its original tissue, recent evidence has established that the dissemination of cancer cells occurs early in primary tumor growth and that cancer cells are shed continuously into the body for eventual colonization of secondary tumors [26,27]. This phenomenon has radical implications for the heterogeneity of CTCs and metastatic cells which must be carefully considered when designing techniques to analyze patient samples and suggest treatment regimens [27].

## 3. Magnetic Transduction

In magnetism, there are typically two poles, positive and negative. Although there is active research in observing a magnetic monopole in nature, the quest to find the particle continues to elude researchers [28]. In two-pole systems, like poles create a repellent force toward one another while opposite poles generate an attractive force. This force follows Coulomb’s law where the magnitude of the force is dictated by how strong the poles themselves are and the distance between them.

(1) $$\vec{F}=k_{e}\frac{q_{1}q_{2}}{r^{2}}\hat{r}$$

Coulomb’s law is shown in Equation (1), where $\vec{F}$ is the force vector, $k_{e}$ is Coulomb’s constant, $q_{1}$ and $q_{2}$ are the signed magnitudes of the two charges, r is the distance between the magnetic sources, and $\hat{r}$ is the unit vector directed along r [29].

### 3.1. Forces on Particles

The magnetic properties of a material are dictated primarily by the electrons which compose the atoms of the material. Most materials have atoms arranged in a random manner, where their respective electrons’ magnetic states cancel each other. The force acting on a magnetic dipole when exposed to an external magnetic field is defined as

(2) $${\vec{F}}_{m}=\left(\vec{m}\cdot \nabla \right)\vec{B}$$

where ${\vec{F}}_{m}$ is the magnetic force, $\vec{m}$ is the magnetic dipole, and $\vec{B}$ is the magnetic flux density [29]. The magnetic dipole is defined by $\vec{m}=V_{m}\vec{M}$, where $V_{m}$ is the volume of the particle and $\vec{M}$ is the magnetization of the material. The magnetic force is related to the differential of the magnetostatic field energy density. This can be further illustrated by defining

(3) $$\vec{M}=\Delta \chi \;\vec{H}$$

$$\vec{B}=\mu_{0}\vec{H}$$

where Δχ  is the effective susceptibility of the magnetic nanoparticle relative to the environment it is placed in, $\vec{H}$ is the applied magnetic field, and $\mu_{0}$ is the permeability constant of free space. When considering that there are no time-varying electric fields or currents in the medium, the equation for magnetic force transforms to

(4) $${\vec{F}}_{m}=V_{m}\Delta \chi \nabla \left(\frac{1}{2}\vec{B}\cdot \vec{H}\right)$$

where the magnetostatic field energy density, $\frac{1}{2}\vec{B}\cdot \vec{H}$, dictates that the resultant force for a particle in a magnetic field is proportional to the strength of the magnetic field, and to the field gradient that the particle experiences. The magnetic flux density gradient can apply a translational force at a distance whereas a uniform field can only apply a torque [30].

### 3.2. Magnetic Torque

A material is classified as ferromagnetic when it has a large number of unpaired electrons throughout its compositional atoms which, when aligned, create a strong unidirectional magnetic field. Because electrons can behave like magnets, a large number of electrons with the same pole orientation creates magnetic domains inside a material. If a ferromagnetic material is applied to the end of a beam fixed by a mechanical flexure on only one side, said cantilever will deflect out-of-plane when exposed to a uniform magnetic field [31]. Magnetic actuators can produce large out-of-plane deflections with high force without the need for onboard power or wiring. This force is generated as the magnetic element torques out-of-plane in the direction of the applied magnetic field. This force is defined as

(5) $$T_{field}=v\vec{H}\vec{M}\sin\left(\gamma -\phi \right)$$

where v is the volume of the magnetic structure, $\vec{M}$ is the magnetization of the material, $\vec{H}$ is the applied magnetic field, and γ−ϕ is the angle between the magnetic field and the magnet.

As such, the deflection achieved is dependent on the strength of the magnetic components, as well as the length, width, thickness, moduli, and angular stiffness of the beam and mechanical flexure [31,32]. The flexure will remain suspended out-of-plane until the magnetic field is removed. Similarly, if the flexure is exposed to a magnetic field of cyclically changing strength, the magnitude of deflection will dynamically adjust as well. This is typically achieved by exposing the actuator device to an electromagnet powered by an alternating current or a rotating/translating permanent magnet.

A fast-moving cyclic magnetic actuator in a fluid will generate a shear force which can disrupt biological masses, such as thrombi [33,34]. However, if an elastic material is attached between the end of the cantilever and the adjacent outer frame of the device, this material will undergo uniaxial stretching as the distance from the cantilever edge and the device’s outer frame changes due to the out-of-plane deflection. For example, Enríquez et al. recently used cantilever magnetic actuators to cyclically stretch fibronectin, a glycoprotein with elastic properties, in an effort to mimic the breathing cycle of the lungs as a platform to study changes in disseminated breast cancer cells upon arrival to the common metastatic location. The material experienced uniaxial stretching as the distance from the cantilever edge and the device’s outer frame changed due to the out-of-plane deflection [35].

Permanent magnets will remain magnetized (remain aligned) after the external field is removed, while other ferromagnetic or ferrimagnetic materials do not have the ability to stay magnetized permanently. Common ferromagnetic materials include iron, cobalt, and nickel. Because the magnetic force is related to the number of electrons which can move, a magnetic field may also be produced by a current of electricity rather than by a magnet [29,36,37].

### 3.3. Thermal Energy

Based on their composition, iron oxide particles are ferromagnetic or ferrimagnetic by nature. In fact, most, but not all, iron oxide nanoparticles are magnetite (Fe_3_O_4_) or maghemite (γFe_2_O_3_), which are ferrimagnetic materials at room temperature [38]. Ferrimagnetic materials below a certain temperature threshold possess the same spontaneous magnetization as ferromagnetic materials. However, a non-uniform arrangement of atomic dipoles is created. Therefore, a lattice often forms of magnetic moments, with a strong magnetic moment directed parallel and a weaker magnetic moment directed antiparallel, leading to a magnetic field still being generated for the bulk material [39]. These materials can achieve magnetically induced heating due to their hysteretic properties when exposed to a time-varying magnetic field. Nevertheless, their heating efficiency is limited due to multiple magnetic domains present in these larger-sized particles.

However, if the diameter of the fabricated iron oxide particle is less than 200 nm, it will exhibit superparamagnetic properties instead. These particles are called superparamagnetic iron oxide particles (SPIOs/SPIONs), with particles under 50 nm being further classified as ultra-small SPIONs [40]. Paramagnetic materials have the same underlying principles as discussed above with ferromagnetic materials, but the coupling of the atomic magnetic moments is small. They do not exhibit a net magnetic moment without an external field and generate only a small magnetic moment when placed within a magnetic field [29]. Therefore, these materials have no magnetic remanence, meaning their magnetization relaxes to zero in a certain amount of time after the removal of the applied magnetic field. The relaxation time relates to either the Brownian relaxation time, the physical rotation of the particle dependent on the surrounding fluid, or the Néel relaxation time, the rotation of the atomic magnetic moments within each particle. The heating mechanisms in magnetic nanoparticles to induce hyperthermia include Neel and Brownian relaxation as well as hysteretic loss. SPION hyperthermia depends strongly on the particle size (<15 nm diameter), where Brownian relaxation exerts thermal energy as the particle rotates and applies shear force against the surrounding fluid, and Neel relaxation dissipates energy as the magnetic moment of the particle rotates before the physical particle [41]. Lastly, superparamagnetic nanoparticles are distinguished as such because they can generate larger field gradients than traditional paramagnetic materials, owning to the field being concentrated on a small particle area [42].

### 3.4. Benefits and Disadvantages of Using Magnetic Forces

A large appeal of generating forces through magnetism is the ability to apply an external magnetic field to direct the movement of a magnetized sample without direct contact or tethering [30,43,44,45,46]. In clinical settings, this has potential for less invasive, targeted therapeutic delivery, although attenuation of the field strength at deep tissue distances must still be overcome, as is similar with penetration of sound, light, and other external stimuli often proposed for non-invasive therapies. In vitro, the contactless, but precise, movement attainable with magnetic force can better preserve the sterility of biological samples and simplifies fabrication within the confines of traditional culturing equipment, such as commercial cell culture well plates, Petri dishes, and incubators, as compared to other force-generating apparatuses (e.g., pneumatics, electrostatic, piezoelectric, etc.) [35].

The equipment used to produce these wireless forces are simple and relatively inexpensive. Permanent magnets are commercially available and electromagnets consisting of wound wire around a high-permeability core can easily be made in the lab. Additionally, the size of the magnetic element is easily scalable, ranging from the size of a gene (single nanometer width) up to the macro-scale. Lastly, due to the diamagnetic nature of most biological materials, there is little interaction or sensitivity of the inorganic magnetic force component with existing cultures, as long as the researcher is mindful of the strength and frequency of the field that is required for a given application [47].

Conversely, the main disadvantage of using magnetic materials in culture is a pronounced cytotoxic effect. Permanent magnets and superparamagnetic particles typically must be coated in a biocompatible material before they can be utilized as a culture platform or a material for cellular uptake. For example, permanent magnets, such as neodymium (NdFeB), may be cytotoxic due to their corrosiveness [48]. Many common coatings are detailed below (see Section 4 and Section 5 and Table 1). However, this is not always sufficient. Ketebo et al. and Shin et al. showed that silica-coated nanoparticles could damage a cell’s cytoskeleton, impairing cell adhesion properties and reducing matrix rigidity/moduli sensing, due to the reactive oxygen species generated [49,50]. Beyond explicit cytotoxicity, all new magnetic particle formulations should undergo verification studies to ensure that they do not alter typical cell metabolism and function once taken up, as this could lead to untranslatable or misleading results [51].

## 4. Introduction to Magnetic Techniques in Cancer Treatment

Numerous magnetic techniques have been developed that show promise for in vivo and ex vivo clinical use. Generally, these techniques allow clinicians to sort, analyze, and/or treat primary and circulating tumor cells by functionalizing SPIONs, which then accumulate at the tumor site (in vivo) or isolate cancerous cells from bulk patient fluid samples (ex vivo). These SPIONs form the basis of many proposed targeted drug delivery treatments, which reduce a patient’s chemotherapeutic burden as the nanoparticles accumulate at the tumor site, enabling a lower systemic dose and a higher local dose [72,73]. SPIONs also encompass a large proportion of proposed hyperthermia treatments, as described in Section 3, where heat is generated from the particles at the tumor site, causing apoptosis when tissue temperatures reach 42 °C and necrosis when temperatures exceed 46 °C up to 48 °C. [74,75,76].

For both treatments, the SPIONs must aggregate to the primary tumor. This is occasionally achieved through a direct local injection, but is more often achieved by external manipulation, where researchers guide the particles using an exterior magnetic field, or through self-aggregation using a tumor-specific antibody-coating on the nanoparticle [74,75,77]. This latter coating also forms the basis of ex vivo magnetic-associated sorting of circulating tumor cells or, occasionally, metastatic cells from patient fluid (e.g., blood, pleural effusions), which can then be analyzed based on cancer cell number isolated, marker expression, genetic profiling, or drug screening assays under the umbrella of precision medicine. Some common antibodies that are conjugated to SPIONs include anti-EpCAM (epithelial-cell-adhesion molecule), anti-HER2 (human epidermal growth factor receptor 2) for HER2+ cancers (which may include breast, bladder, pancreatic, ovarian, gastric, and other cancers), and anti-CD63 (blocks phagocytosis and is commonly used to help identify extracellular vesicles). Antibodies may be conjugated for negative magnetophoresis as well, such as anti-CD45 to remove leukocytes from bulk patient samples [78,79,80,81,82,83,84,85]. Additional common SPION coatings include non-specific proteins or polysaccharides (e.g., serum albumin, dextran, chitosan) or hydrophilic inert polymers (e.g., polyethylene glycol, polyvinyl alcohol). These coatings improve biocompatibility and aqueous colloidal stability, while decreasing opsonization in the bloodstream and uptake of the particles by off-target cells, such as macrophages [73,77].

Some SPION formulations have already gained FDA approval or are currently in clinical trials [86,87,88,89]. For example, magnetic hyperthermia for cancer was introduced to clinical practice in 2011 when it was approved for the treatment of glioblastoma (as a combination treatment) [77]. Due to the breadth of research in this area and the advanced stage of clinical translation, these magnetic nanoparticles have been well-reviewed in the literature. It is important to note, however, that there may still be significant challenges to overcome for many of these SPION techniques as clinical data becomes available. For example, although several SPION-based platforms have been approved as contrast agents for magnetic resonance imaging (MRI), multiple have since been withdrawn from the market due to insufficient clinical trial results and/or major safety concerns that emerged [90]. We direct the reader to several modern and excellent reviews on SPION use for cancer treatment for further information [75,77,90]. Here, we will instead focus on recent magnetic techniques used primarily in vitro that enable researchers to probe questions on cancer cell behavior through the metastatic cascade that cannot be appropriately ascertained from in vivo experiments.

### Why Use In Vitro Magnetic Techniques to Study Disease Progression?

Studying certain aspects of the metastatic cascade requires in vitro investigation, which allows for a reduced number of variables and an ability to gather real-time data of cell responses to precise stimuli. For example, in vitro experimentation is crucial for mechanotransduction analyses of cancer cells, because cell response can be systemically observed against changing substrate topographies, moduli, and mechanical (i.e., tensile and contractile) movements in a decoupled way that in vivo studies of metastatic progression cannot resolve. In this way, building in vitro biomimetic devices can also enable researchers to more easily study the behavior of small populations of disseminated cancer cells upon arrival to each premetastatic niche, before the cells would reach a mass sizable enough to be traditionally detected in vivo. Beyond the detectable size limitation, evaluating disseminated cells within an expanding and contracting tissue, such as within the lungs of the animal, would be an extreme technical challenge in vivo and, if possible, would likely cause a great deal of animal suffering due to the invasive constraints that would be required. Lastly, in vitro models can provide benefits to researchers by reducing the time and cost of most experiments [91,92]. Because of these benefits, multiple in vitro techniques have been developed, including scaffold-free spheroids in suspension as well as scaffold-based hydrogel embedding and matrix stretching.

For many of these culture platforms, magnetic techniques have been proposed to improve the reproducibility of the in vitro and ex vivo experiments. By incorporating cells with magnetic tags (e.g., magnetic beads, magnetized media), greater sample homogeneity is suggested, for example, in more precise patterning of cells onto a substrate or more reproducible geometries of cells than traditional clustering methods [93]. We will examine each of these proposed magnetic-based techniques that can be used to resolve behaviors of cancer cells related to these hard-to-observe aspects of disease progression.

## 5. Magnetic-Directed In Vitro Cell Aggregation

Multiple studies have established that cells cultured in 2D do not exhibit the same response to stimuli, such as chemotherapeutics, as what is observed in vivo. For example, cancer stem cells do not appear to survive extended 2D culturing, while 3D culturing preserves the stem-like phenotype associated with self-renewal and asymmetric division of the tumor sample. Because these cancer stem cells are thought to be a leading cause of treatment resistivity and eventual relapse, their preservation is crucial for translatable results, such as high-throughput drug screening [94]. Three-dimensional culturing is now commonly used to more accurately observe cell behavior during in vitro experimentation. 3D culture techniques are broadly categorized as scaffold-based or scaffold-free, where the scaffold is defined as a supporting matrix or substrate that the cells can attach to and which facilitates the multi-layered depth of the culture [95,96].

The aggregation of cells into spheroids is a particularly common scaffold-free technique in cancer research. ‘Spheroids’ generally refer to cells taken from a 2D monoculture, where they were initially expanded, and cultured in suspended media. This suspension drives the aggregation of the free-floating cells into sphere-like clusters dominated by cell-cell attachments, such as N-cadherin and E-cadherin interactions [96,97]. Traditional techniques for spheroid formation include the hanging drop and liquid overlay methods, as well as the use of spinner flasks. However, there are several challenges to culturing spheroids with these techniques that have greatly limited the use of spheroids for accurate, translatable research in cancer. Namely, spheroids formed with these methods tend to lack uniformity and reproducibility with respect to aggregate geometry and packing density [94,97]. This is especially concerning for applications such as high-throughput drug screening, where the mass transport rates of the chemotherapeutics may vary between wells such that an accurate comparison of drug sensitivity is not achievable [98]. Additionally, traditional spheroid formation techniques present extra challenges whenever reagents have to be replaced during culturing, such as for media changes or immunostaining, due to the free-floating nature of the aggregate. Incorporating magnetic forces into spheroid culture platforms address many of these obstacles, as described below. Magnetic forces are added to the spheroid culture either with a set of permanent magnets sandwiching the culture plate or with a single magnet either above or below the culture dish. These setups are described in Section 5.1 and Section 5.2 and summarized in Table 1 below.

### 5.1. Magnetic Levitation (Dual Magnet)

**Figure 1:**
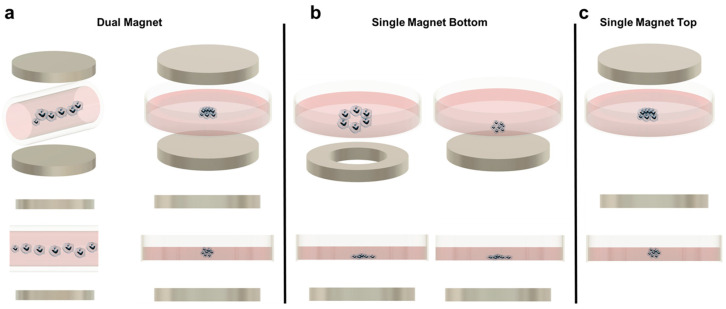
(**a**) Magnetic levitation with dual magnet configuration for cell patterning. Left: Dual magnet using a capillary tube to create a row of spheroids. Right: Dual magnet using a Petri dish to levitate cells. (**b**) Single magnet bottom patterning. Left: Ring magnet in the bottom for cell patterning leaving a void in the middle. Right: Single magnet bottom arranging the cells flat in the bottom of the Petri dish. (**c**) Single magnet top patterning.

In magnetic levitation, cells with internalized paramagnetic beads are suspended in media due to a magnetic field created by two permanent magnets placed on the top and bottom of the culture plate (Figure 1a). In this way, the aggregates levitate to a certain equilibrium height based on the balance of magnetic, gravitational, and buoyancy forces [52]. Magnetic levitation may occur with magnets sandwiching individual wells, where each well will form one spheroid, or sandwiching the length of a capillary channel, where multiple spheroids will form along the tube, sharing culture media [52,53]. Additionally, a paramagnetic media (media containing a known concentration of paramagnetic agent) can be used to suspend the cells rather than incorporating paramagnetic beads [53,54].

Although magnetic levitation is promising, the technique must still overcome several limitations. One issue involves light and fluorescent microscopy. In principle, magnetic levitation improves kinetic imaging abilities because the spheroids can be spatially manipulated without direct contact and these spheroids should not undergo translational movement after reaching equilibrium. Successful traditional imaging has been demonstrated in a few studies [52,53,57]. However, for many magnetic levitation platforms, only side-view images are accessible due to the sandwiched magnets around the dish, which are not compatible with standard microscope objective positioning. A second limitation may be the increased cost associated with magnetic levitation over traditional spheroid-forming techniques, due to the additional paramagnetic beads or soluble paramagnetic agents that must be manufactured and incorporated into each sample [94].

### 5.2. Magnetic Patterning and Single Magnet Levitation

Aggregation patterning, often called magnetic bioprinting or magnetic micropatterning, typically refers to a magnetic force concentrated underneath the culture area which can force the cells into an aggregate as in 5.1, or into additional, distinct 3D geometries (Figure 1b). Occasionally, though, these techniques are used to spatially pattern 2D cultures. For example, Paun et al. fabricated a checkerboard pattern where the squares were composed of a photopolymer with or without SPIONs. When the substrate was not exposed to a magnetic field, seeded fibroblasts spread throughout both areas in a traditional monolayer. However, when a static magnetic field was produced using permanent magnets underneath the device, the fibroblasts were only observed on the squares with magnetic nanoparticles, demonstrating a proof-of-concept patterning of cells into specific culture areas [99]. Fu et al. similarly magnetized polyethylene glycol-diacrylate and used it as a removable block to pattern cells into specific shapes. This technique was also used to pattern multiple cell types sequentially, by first allowing cells to attach in the areas surrounding the hydrogel, and then allowing a new cell type to flood the geometry of the hole created once the hydrogel was removed [100].

Concerning the creation of cell aggregates using a single magnetic source, a few setups have been developed. Similar to dual-magnet magnetic levitation, these can rely on the use of a paramagnetic media or, more popularly, paramagnetic nanoparticles taken up by cells before aggregation [55,62,67,69]. Additionally, aggregates can still be assembled in individual wells using a permanent magnet underneath each well bottom, or multiple aggregates can be assembled into an array in a large Petri dish such that they share media [55,63]. In the latter, the magnetic force is generated by an equivalent array of magnets, such as the pin-holder device developed by Dr. Hiroyuki Honda’s laboratory. The pin-holder device is a block of magnetic soft iron in which material was removed from the top surface to create more than 6000 free-standing rectangular prism pillars [69,101,102]. Because the spheroids share media, this technique cannot be used to study the effect of different conditions, such as how different concentrations of chemotherapeutics affect cell viability in a high-throughput drug screen. However, the spheroid interaction does allow for other investigations, such as tracking the migration/invasion of cells from one aggregate to another and the formation of vascular networks when spheroids are embedded into a collagen type I hydrogel [70,71].

Aggregate assembly using a magnetic source only underneath the culture area provides several advantages. It is the simplest when considering longer culture times or more complex experimental designs. This is because the device is not reliant on a magnet above the culture plate, so the forces at equilibrium in the culture are not disturbed when the lid is removed for media changes or reagent additions. In addition, because the aggregates are only forced downward, they are more susceptible to the shape of the magnet used. Researchers have taken advantage of this by using ring magnets instead of solid magnets, to create lumens (Figure 1b). These constructs are primarily associated with tissue engineering applications but may prove useful for cancer research. For example, Timm et al. proposed the use of the 3D ring structure to quantify 3D cell toxicity against varying concentrations of a drug. Once the ring structure was formed and the drug was added, the permanent magnet was removed, and the rate of ring closure was quantified as a function of the concentration of the drug added. Although this particular study did not utilize cancer cells and a chemotherapeutic drug, the technique could easily be adapted for such a purpose [58,60,61,68].

The main disadvantage of using a single magnet below the culture dish is the geometry of the aggregate formed. Because the aggregate is not suspended (except in cases where the cells are embedded in a hydrogel), a complete spheroid is often not created. Instead, most aggregates resemble half-spheroids (or less discernible shapes) which have a flat bottom against the culture plate, although this is a function of the magnetic field strength used (Figure 1b). Additionally, most microscopes used in biological experiments are inverted. Therefore, having the magnet below the dish makes this platform incompatible with the microscopes that most cell culture labs will have readily available, requiring upright microscopes instead. To mitigate these two issues, some researchers have developed single-magnet levitation platforms, where one magnet is placed above the culturing area (Figure 1c). Kim et al. developed a system consisting of a magnet on the culture plate lid with a magnetized iron pin protruding underneath that focuses the magnetic field. This focused magnetic field, coupled with magnetic nanoparticles in the cells of interest, results in a uniform and reproducible spheroid geometry [56].

In 2010, Dr. Glauco R. Souza et al. used gold and magnetic iron oxide particles in a bioinorganic hydrogel composed of bacteriophage to develop a magnetic levitation platform that utilized one permanent magnet above the culture dish [57]. The colleagues founded the company, Nano3D Biosciences (n3D), for the magnetic drive system (with the technology now under Greiner Bio-One) that features various arrays of neodymium magnets for high-throughput culturing [103,104]. The particle manufacturing was also patented and marketed as NanoShuttles (and today includes the product NanoShuttle-PL for their gold, iron oxide, and poly-L-lysine particles as well). These products have been used by many research groups over the past decade for numerous applications, including multi-cell cancer spheroid generation, drug screening, tissue engineering, and primary ex vivo cultures of cancerous cells [59,64,65,66]. Due to its widespread use and industry support, an inclusive culture experience has been developed, with established protocols to first bioprint the magnetized cells with the magnetic drive underneath the culture area, then to position the magnetic drive on top of the platform to levitate the cells into spheroids. The magnetic drive also moves underneath the plate to facilitate media changes and accessory products have been developed to further simplify spheroid-based assays. These include the magnetic pen, which enables easy handling of the spheroid if it needs to be moved between wells [65]. This system, in addition to the previously described techniques, demonstrates the clear advantages in the precise creation and movement of in vitro 3D cell aggregates that can be achieved by the simple addition of magnetic force.

## 6. Magnetic Techniques to Probe the Cell and Its Microenvironment

A subset of common in vitro magnetic techniques involves the use of antibody-functionalized magnetic beads to sort a desired population out of a bulk culture. These include using magnetic associated cell sorting (MACS), isolating extracellular vesicles released by cancerous cells, and pulling down DNA, RNA, or specific proteins from lysed cells for quantification [105,106,107,108,109,110,111]. Magnetic particles have even been proposed as a method to improve transduction efficiency in conjunction with standard lentiviral particle use or instead of it [112,113,114]. These techniques often follow the particle principles outlined in Section 3 and Section 4. In this section, we will instead focus on magnetic techniques that resolve the mechanical properties of cells and their environment or illustrate cells’ biological response to dynamic mechanical stimulation.

### 6.1. Extracellular Matrix (ECM) Patterning and Detection of Remodeling

**Figure 2:**
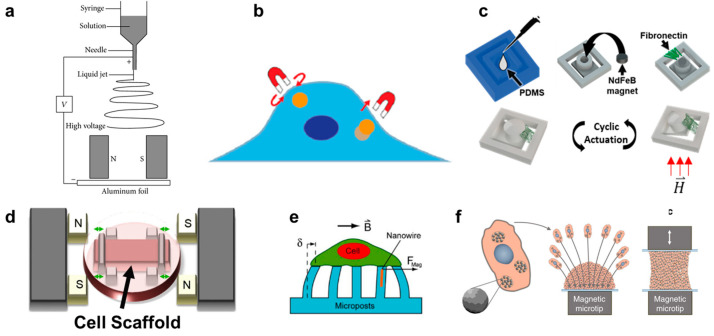
(**a**) Schematic depicting magnetically assisted electrospinning to create aligned matrix fibers [119]. (**b**) Schematic depicting how magnetic tweezers interact with the magnetic nanoparticles inside a cell [120]. (**c**) Fabrication process of fibronectin-coated polydimethylsiloxane (PDMS) cantilever with embedded permanent magnet. The cantilever deflects once exposed to a magnetic field enabling cyclic actuation [35]. (**d**) Uniaxial stretching platform using magnetic posts to stretch a cell scaffold [121]. (**e**) Magnetically active microposts to induce forces on cultured cells [46]. (**f**) Magnetic nanoparticle embedded cells for remote mechanical control. Magnetic microtips can aggregate the cells to create a tissue [45].

In contrast to the scaffold-free spheroid formation discussed in Section 5, scaffold-based 3D cell cultures are composed of cells seeded onto a supporting matrix. This matrix may be a gellable polymer, such as collagen, Matrigel (naturally derived), or polyethylene glycol (synthetic) [115]. The gelation process is typically simple and enables cells to be cultured on top of or throughout the gel. However, researchers may choose to undergo additional processing steps with their matrix of interest in order to create alternative scaffolding. One common process is called electrospinning. In this technique, a viscous liquid is drawn into a fiber which continuously builds on a collection plate until a complete mesh is fabricated [116]. In standard electrospinning, the fibers of the mesh are aligned and layered in a random orientation. However, using different mechanical, electrostatic, or magnetic interventions, the alignment of the electrospun fibers can be more precisely controlled. In magnetic-assisted electrospinning, an external magnetic field is generated through two parallel permanent magnets (Figure 2a). The fibers, drawn out of solution by standard electrospinning, will be driven to align parallel to the magnetic field lines as they travel toward the collection plate. This technique requires that the material being spun responds to the magnetic field generated. This can be achieved by incorporating one of the numerous nanoparticles into the matrix, such as silver nanorods, carbon nanotubes, or superparamagnetic nanoparticles [116,117,118].

In addition to magnetic-assisted electrospinning, Kim et al. recently demonstrated that ECM proteins could be chemically crosslinked onto magnetic particles and self-assembled into numerous topographical patterns in a surrogate hydrogel by adjusting the external magnetic field applied [122]. Martin et al. incorporated iron oxide nanoparticles into nonmagnetic materials, such as silica and calcium phosphate, and developed an SLA-based 3D magnetic printing protocol. To demonstrate the technique, the researchers aligned an external magnetic field with respect to different axes and observed changing bulk mechanical properties of the printed device based on the orientation of the reinforcing elements of the discontinuous fiber composite [123,124]. Margolis et al. similarly demonstrated that the mechanical properties of alginate could be altered by loading the hydrogel with magnetic nanoparticles of various sizes and concentrations and exposing the system to an external magnetic field. The resulting gel formed an aligned microporous structure with anisotropic topical features and stiffnesses related to the direction of the magnetic field, which ultimately dictated the morphology of mouse myoblasts cultured within [125].

Lastly, a magnetic technique was developed to selectively identify ECM remodeled by cancerous cells in an in vitro tumor-like coculture environment [126]. Magnetic helical nanorobots were fabricated such that contactless forward or backward movement could be achieved when the devices were subjected to a rotating magnetic field. The nanorobots were made from silica with embedded iron particles. The robots were injected into a hydrogel of basement membrane protein with breast cancer cells and non-cancerous breast epithelial cells cocultured together. The robots were injected on one side of the hydrogel and driven across its length for approximately 30 min using an external magnetic field. The microrobots preferentially attached to ECM surrounding the cancer cells, with comparatively few microrobots found around the healthy cell type. This phenomenon seems driven by the difference in charge surrounding cancerous and healthy cells, where microrobots became irreversible stuck when passing up to 35 µm away from a cancer cell, but only up to 15 µm away from a healthy cell. This charge difference is driven by a sialic acid linkage aberrantly expressed in the cancer cells, which imparts a large negative charge on the surrounding ECM. At present, the experiment was only performed with different breast cancer cell lines in the reconstituted basement membrane protein [126]. Although sialylation changes have been reported in a number of different cancer types, it remains to be seen if the designed microrobots will retain the same preferential attachment for other cancer lines and/or in other matrices based on their unique ECM remodeling [126,127]. It is also of interest if the results would be replicated in environments with more cell complexity, as fibroblasts, not breast cancer cells, seem to be the main source of ECM remodeling in the tumor microenvironment [128].

### 6.2. Resolving Dynamic Mechanotransduction Behavior

Mechanotransduction is the process by which cells convert mechanical stimuli into biochemical signals [129]. This mechanical stimulation can include all aspects of the surrounding physical environment, including its moduli and topography, as well as dynamic compressive, tensile, or shear forces acting on the cells [120]. Cells have several mechanisms through which they detect mechanical stimulation. The surface of the cell itself has mechanical sensitive protein complexes, mechanosensitive ion channels, and transmembrane integrins which connect the intracellular cytoskeleton to the ECM [129,130]. Mechanoresponsive proteins also exist within the cell, typically as a downstream effect of a membrane mechanosensitive signal being triggered (e.g., integrin signal). The conformation of these protein complexes will transition in a mechanically stimulated environment (e.g., when the cell is under tension) which will alter the resulting binding properties or enzymatic function [129].

Using magnetic techniques to deliver mechanical stimulation to cells is very attractive due to its potential to be contactless, simple to fabricate, and easily integrated within current culturing equipment, as mentioned in Section 3.4. In addition, magnetic nanoparticles can be effectively utilized to deliver targeted, non-destructive mechanical stimulation to cells, or even directly to the surface mechanoreceptors of cells. In this way, some magnetic techniques offer the unique ability to mechanically load cells on soft or fragile biomaterials since no extracellular scaffold deformation is required [129]. In this section, we will highlight different magnetic devices that can provide dynamic mechanical stimulation either directly to cellular components or to the outside of the cell (i.e., scaffold deformation) and how these stimuli affect cell function.

#### 6.2.1. Applying Mechanical Force Intracellularly

Magnetic pulling cytometry and magnetic twisting cytometry using magnetic tweezers are the main magnetic techniques available to measure intrinsic mechanical properties of cells [131] (Figure 2b). For these techniques, magnetic beads are typically incubated with cells in culture to allow for cellular uptake. In magnetic pulling cytometry, a magnetic needle is then positioned close to the magnetic bead in an isolated cell, such that the bead is pulled in-plane toward the needle with a known force. In magnetic twisting cytometry, a strong external magnetic field is first pulsed (usually ≥1000 G for <0.5 milliseconds) which magnetizes the bead in the direction of the magnetic field. Then, a weak twisting field is applied in the orthogonal direction to the magnetic moment of the bead, causing the bead to attempt to deflect out of plane, similar to the principles of magnetic actuation [120,132,133,134]. Magnetic manipulation of the internalized beads has revealed several intrinsic mechanical properties of cells, such as cytoplasm viscoelasticity [131,135]. However, this technique has also been used to generate intracellular force long-term (≥hours) and observe the resulting cellular changes. For example, Qiu et al. demonstrated that prolonged intracellular force generated by internalized magnetic beads would result in the alignment of F-actin fibers in the direction of the magnetic force in endothelial cells [136].

Additionally, these techniques can also be used to measure the force of or dynamically manipulate specific cell receptors. For example, Boulter et al. used fibronectin-coated nanobeads and magnetic tweezers to uncover crosstalk between integrin rigidity mechanosensing and cellular metabolism. They found that the integrin coreceptor, CD98hs, indirectly regulated sphingolipid synthesis after mechanical integrin disturbance through the prevention of several upstream regulators of RhoA, such as Src kinases [137]. Others have used numerous coatings, including for the RGD domain (to target integrin α_v_β_3_), anti-β1 integrin, anti-PDGFRα, E-cadherin extracellular domains, and the extracellular loop of TREK-1 [133,135,138,139,140,141,142,143,144].

The main disadvantage to these techniques is that they are often extremely costly and technically challenging. These challenges are not unique to magnetic-based approaches (e.g., optical tweezers), but are a common issue associated with being able to stimulate and then observe minute intracellular forces [145]. Specifically for magnetic-based approaches, both finely calibrated magnetic field control equipment and extremely uniform magnetic beads are essential for accurate measurements [120]. Additionally, an overarching obstacle for the field of intracellular mechanotransduction is a lack of consensus between experimental techniques. Specifically, Wu et al. measured the MCF-7 breast cancer cell line using six techniques (atomic force microscopy, parallel-plate rheology, optical stretching, cell monolayer rheology, magnetic twisting cytometry, and particle tracking microrheology) and demonstrated that the obtained elastic and viscous moduli of the cells varied up to 1000- and 100-fold, respectively [135]. This inconsistency must be carefully considered as mechanosensitive cellular pathways become increasingly investigated by various techniques and research groups, such that our collective understanding of mechanotransduction throughout the metastatic cascade can continue to grow.

#### 6.2.2. Extracellular Movement

Although static cultures have provided invaluable insights into metastatic progression, they fail to capture the response of cancer cells to dynamic extracellular forces, such as forces that are native to a distant organ on early disseminated cancer cells. Here, will we discuss magnetic techniques used to apply dynamic and static forces extracellularly, encompassing compressive, traction, and tensile forces.

The growth of a carcinoma is associated with tremendous compressive forces within the bulk of the tumor as cells, ECM, and fluid accumulate. Compressive forces also increase within the surrounding microenvironment as the growing tumor pushes against the boundaries of healthy tissue. In an unusual technique, Fernández-Sánchez et al. developed a method to generate compressive forces in vivo. Magnetic liposomes or ultra-magnetic liposomes (i.e., liposomes loaded with a magnetic aqueous fluid or loaded with superparamagnetic iron oxide nanocrystals, respectively) were fabricated in vitro and then intravenously injected in mice. Then a permanent disc magnet was inserted subcutaneously in front of the colon. After one week, the ultra-magnetic liposomes were concentrated in the stromal cells surrounding the distal colon crypts and remained with a stable concentration of iron per gram of tissue over a one-month span. The compressive force, and subsequent pathophysiological stress on the mesenchymal cells, was consistent with the mechanical pressure exerted by early tumor growth in adjacent crypts as measured in previous cancerous mouse models. Their subsequent biological investigation indicated that tumorigenic pathways may be activated in the non-cancerous stroma surrounding a carcinoma due to the compressive mechanical stimulation, which could contribute to an unstable positive feedback loop between oncogene expression and tumor induction [146].

In vitro, several devices have been built which can provide dynamic compressive and tensile forces onto cells on polymer substrates. In one system, magnetic actuators transmitted linear uniaxial compressive or tensile forces through positioning pins to a cell culture area, consisting of cell-seeded polyethylene glycol constructs. Although the entire device was sealed for sterility, it was built around one culture area, such that it cannot be easily upscaled for high-throughput assays [147,148]. In another system, a magnetic force was created between an external electromagnet and a permanent magnet embedded into a polydimethylsiloxane (PDMS) frame. The PDMS frame was fixed on one side and allowed to freely move toward the electromagnet on the other. In the middle of the PDMS frame was a mesh where a thin layer of Matrigel was deposited and cells were subsequently seeded to undergo cyclic stretching [149]. Enríquez et al. developed a magnetically actuating device along a similar principle (Figure 2c). Here, the matrix protein, fibronectin, was suspended between the cantilever and adjacent frame edge of a PDMS magnetic actuator, resulting in cyclic stretching of the matrix (and cells within) as described in Section 3. A main advantage of this recent design is the suspended matrix, which does not utilize any polymeric support mesh in the culture region of interest (Figure 2d). It also benefits from a high-throughput design, consisting of an array of permanent magnets in a linearly moving actuating platform that sits beneath a standard multi-well culture plate [35].

Lastly, similar devices have been developed to those described above, but which solely use PDMS gel as the culture substrate [150]. A unique subsection of this category is magnetic micropillars (Figure 2e). Cells are cultured on top of magnetized PDMS pillars, such that the bottom of the cell will undergo tractile forces as the pillars bend toward an external magnetic source. The pillars are typically magnetized by iron-coating over the PDMS or by embedding magnetic nanoparticles into the polymeric pillars [151,152,153]. Like magnetic tweezer techniques, these distinct pillars can be used to isolate and quantify forces at individual cell-matrix contact sites due to their free motion from one another. Additionally, this magnetic method is one of few that can detect traction forces that cells impose onto the substrate during migration [154]. However, the translation of these results to in situ cell response is likely to suffer due to the lack of ECM proteins used in the culture platform as well as the often unrealistic stiffness of the PDMS compared to the native tissue of the cell type. In contrast, Du et al. developed a magnetic tissue stretcher that utilizes no matrix or substrate at all. This technique begins identically to aggregation micropatterning, where magnetic nanoparticles are taken up by cells which are then forced to aggregate due to a magnet underneath the culture area (a glass slide) (Figure 2f). However, a second magnetic microtip-glass slide apparatus is then placed in contact with the top of the aggregate, and these cells are allowed to attach. The confluent band of tissue can then undergo cyclic tensile strain driven by the movement of one of the magnetic microtips. In this way, although the cells at the very edges are influenced by the topography and modulus of the glass slides, the bulk tissue sample is free of any substrate [45]. Because no in vitro system can replicate all aspects of the in situ tissue environment (nor is it meant to), we will likely find that it is a combination of scaffold and scaffold-free mechanotransduction techniques which together allow us to decouple different aspects of the metastatic cascade and eventually understand the complete and complex process.

## 7. Conclusions

The use of magnetic forces can greatly improve our ability to diagnose, treat, and fundamentally understand cancer. Magnetic nanoparticles are being widely explored for in vivo applications, including as MRI contrast agents, hyperthermic treatment agents, and targeted drug delivery vehicles [38,73]. They also form the basis of most ex vivo cell isolation techniques, such as for isolating circulating tumor cells, wherein antibody-conjugated SPIONs are used for negative or positive magnetophoresis. Here, we focused on in vitro culture platforms that utilize magnetic force to broadly (1) improve the reproducibility and ease of handling of spheroids and (2) impose mechanical force intracellularly and extracellularly. The latter allows researchers to understand the mechanotransduction of cancer cells throughout several unique steps of the metastatic cascade, such as during primary tumor growth or early dissemination. Although using magnets comes with several advantages, such as the untethered transmission of force, the potential effect of large magnetic fields on cells cannot be overlooked in these culture platforms. Both a prolonged strong magnetic field without nanobeads and the unstimulated magnetic nanobeads have been shown to affect proliferation rates, cell metabolism, ion channel activity, and the cytoskeletal organization in a small subset of experiments [49,50,155,156]. The inherent biological effect of using external magnetic forces must be especially accounted for when analyzing certain types of cancers, such as when using cancerous neural cells [157]. With the proper consideration and controls, magnetic force can be an invaluable addition to in vitro culture platforms for reliable probing of cancer cells throughout primary tumor growth and metastatic progression.

## Figures and Tables

**Table 1:** Magnetic-based 3D cell aggregation.

Magnetic Agent Used with Cells	Magnet Type	Type of Aggregation Assembly	Notes	Source
Cadolinium(III) chelates	Magnetized media	Dual magnet levitation	Multiple spheroids share media within a capillary tube	[52]
Gx				[53]
Paramagnetic metal halides				[54]
Gadopentatic acid (Gd-DTPA)		3D magnetic patterning		[55]
Magnetite nanoparticles isolated from magnetic bacteria	Internalized iron oxide nanoparticles	Single magnet levitation		[56]
Magnetite (Fe_3_O_4_), gold and bacteriophage nanoparticles (NanoShuttle)			Ring magnet [57,58]	[57,59]
NanoShuttle-PL				[58]
		n3D magnetic drive system	Cell lumen formed [60,61]	[60,61,62,63,64,65,66]
Magnetite nanoparticles with bovine serum albumin coating		3D magnetic patterning		[67]
Magnetite nanoparticles			Spheroids formed by hanging drop. Spheroids then patterned into lumens using magnetic patterning	[68]
Magnetite nanoparticles in liposomes	Internalized iron oxide cationic liposomes		Cells cultured in media and in collagen I	[69,70,71]
			Multiple spheroids share media	

Each published system uses a combination of permanent magnets outside the culture area and a magnetic agent incubated with cells to improve the tunability, reproducibility, and precise patterning/movement of spheroids. Spheroids were composed of a variety of cell types, including non-transformed cells (e.g., fibroblasts and endothelial cells), various cancer cell lines (e.g., MCF7 (breast), MDA-MB-231 (breast), HCC827 (lung), DT66066) (pancreatic)), and co-cultured spheroids consisting of multiple cell types. These systems have been categorized based on the type of magnetic agent used and the type of levitation/patterning that the device achieves, with notes specifying if the system deviates from the standard practice of forcing cell aggregation into a single spheroid in a media bath.
